# Deep Learning Based Emotion Recognition and Visualization of Figural Representation

**DOI:** 10.3389/fpsyg.2021.818833

**Published:** 2022-01-06

**Authors:** Xiaofeng Lu

**Affiliations:** Department of Fine Arts, Shandong University of Arts, Jinan, China

**Keywords:** deep learning, emotion recognition, graphic visualization, neural network, CNN-BiLSTM

## Abstract

This exploration aims to study the emotion recognition of speech and graphic visualization of expressions of learners under the intelligent learning environment of the Internet. After comparing the performance of several neural network algorithms related to deep learning, an improved convolution neural network-Bi-directional Long Short-Term Memory (CNN-BiLSTM) algorithm is proposed, and a simulation experiment is conducted to verify the performance of this algorithm. The experimental results indicate that the Accuracy of CNN-BiLSTM algorithm reported here reaches 98.75%, which is at least 3.15% higher than that of other algorithms. Besides, the Recall is at least 7.13% higher than that of other algorithms, and the recognition rate is not less than 90%. Evidently, the improved CNN-BiLSTM algorithm can achieve good recognition results, and provide significant experimental reference for research on learners’ emotion recognition and graphic visualization of expressions in an intelligent learning environment.

## Introduction

The rapid development of artificial intelligence (AI) (Ustun et al., 2021), big data (Wang J. et al., 2020), and Blockchain technology (Lv et al., 2021a) has changed the social structure, talent demand, as well as the form of social education. Through traditional data acquisition methods, people need a lot of time and energy to collect data, which hinders the convergence and synchronization of art developed to a certain extent. With the rapid development of information technology, in the internet era, art information and exhibition information around the world can be known by global users in a very short time. People can easily collect landscape materials from all over the world online without leaving home.

Meanwhile, the computer gave birth to new art forms and ideas. Through the computer, the scope of traditional art expression has also expanded from oil painting, traditional Chinese painting, printmaking, sculpture, watercolor, etc. to animation art, image art, photoelectric art, etc. through the sketches drawn by artists. The change in technologies has caused the innovation of the learning environment, and the intelligent learning environment with the Internet of Things (IoT) technology as the core has begun to attract extensive attention from people. In the intelligent learning environment, teachers carry out teaching activities online through the Internet, and learners can easily acquire and learn knowledge through the network. However, psychological research has shown that various emotions generated in the learning process can affect the learning effect. For example, positive emotions such as happiness and satisfaction generated in the learning process are conducive to raising learning interest, while emotions such as boredom and anxiety can hinder the cognitive process. In traditional teaching activities, face-to-face communication between teachers and students enables learners to maintain a positive interest in learning at any time. In contrast, it is difficult for teachers and students to feel each other’s emotional state in time due to the constraints of time and space in the intelligent learning environment. Correspondingly, it is urgent to seek out an effective way to combine knowledge transmission with emotional communication in the current intelligent learning environment.

Human emotions are complex and simple. As a smart species currently in the dominant position on the earth, humans can express emotions through various methods, such as voice, text, and facial expressions (Jain et al., 2019; Khare and Bajaj, 2020; Guanghui and Xiaoping, 2021). In the intelligent learning environment, emotion recognition of learners’ images in class hours through computers and deep learning algorithms can facilitate timely monitoring of psychological and emotional states of learners. The emotion recognition through facial expression images requires high-quality cameras to capture facial images, resulting in high implementation cost. Therefore, the speech-based human emotion recognition method has gradually become the principal method to study human-computer emotion recognition. In the process of communication and expression, speech of humans not only contains semantic information, but also implies rich information like the speaker’s emotion. Therefore, the research on emotion recognition based on human speech and image through computer and intelligent algorithms of deep learning is of great significance.

Speech-based emotion recognition (Liu and Fu, 2021) has been using the method of acoustic statistical features since it was proposed in the 1980s. Until the 21st century, the fast-growing computer multimedia technology and the continuous breakthrough in the field of AI technologies have made great progress in speech-based emotion recognition. The traditional machine learning algorithms based on Gaussian mixture model (Tian et al., 2020), support vector machine (SVM) (Chuan et al., 2020), and artificial neural networks (Shao et al., 2020) have achieved brilliant results in speech-based emotion recognition tasks. However, the traditional machine learning algorithms have some defects in the accuracy of emotion recognition by speech and images. Improving the accuracy of emotion recognition by speech and images based on existing technologies is a critical goal of AI and deep learning algorithms.

The traditional speech-based emotion recognition database can only reach 84.3% for human speech recognition. To ameliorate the shortcomings of traditional machine learning algorithms in speech and image recognition accuracy and enhance the accuracy of emotion recognition, the innovations of this article are as follows:

(1) The unsupervised adversarial autoencoder is used for feature extraction.
(2) Both speech and images are taken as carriers to carry out emotion recognition through the convolutional neural network (CNN).
(3) The neural network is used to collect the information and perform visualization of the learners’ facial expressions.
(4) A convolutional neural network-Bi-directional Long Short-Term Memory (CNN-BiLSTM) algorithm is used to analyze the emotion through speech and images of learners in the intelligent learning environment. The machine learning algorithm based on classification enhancement is used for speech emotion recognition. Compared with the traditional speech-based emotion recognition algorithm, the CNN-BiLSTM algorithm reported here has higher accuracy and can achieve better recognition results.

## Recent Related Works

### Deep Learning and Emotion Recognition by Speech and Images

As the computer technology develops, online education through the internet has become an upsurge of the new era. For example, Kim and Kim (2021), through the research on the user experience of the online education project of primary schools and art galleries, suggested that the user experience satisfaction of the online art education project of primary schools was higher. Budur et al. (2021) discussed the online education and learning among students during Corona Virus Disease 2019 (COVID-19) and showed that online education had become a new educational trend. With the vigorous development of AI technology, there are many works on deep learning in speech and image recognition. At present, emotion recognition technologies by speech and images have gained some achievements. For example, Hossain and Muhammad (2019) applied the deep learning and cognitive wireless framework to the audio-visual emotion recognition system that could automatically identify patients’ emotions in the Internet medical care framework. They evaluated the system through experiments and proved that the system was beneficial to the development of Internet medical care. Khalil et al. (2019) summarized the research on speech-based emotion recognition using deep learning technology, and expatiated the deep learning technology of speech-based emotion recognition. The authors performed simulation of multimodal emotion recognition, and the experimental result demonstrated that the data could be input efficiently by audio-visual and other means. Mellouk and Handouzi (2020) studied the recent works of automatic facial emotion recognition through deep learning. They found that related scholars focused on exploiting technologies to explain and encode facial expressions and extract these features to accomplish excellent forecasts by computers. Their research results showed the effectiveness of deep learning algorithms. Masud et al. (2020) studied intelligent face recognition based on deep learning in the Internet of Things and cloud environment, and compared the performance of this method with the most advanced face recognition depth model. The experimental results indicated that the accuracy of the proposed model could reach 98.65%. Li et al. (2021) validated performance of medical image fusion model based on deep learning algorithm. They found that the deep learning model could automatically extract the most effective features from the data, and it could enhance the efficiency and accuracy of image processing when used for image fusion. At the same time, increasing the scale of training data could further improve the training accuracy. Xiong et al. (2021) explored plant phenotypic image recognition based on deep learning technology, and adopted CNN, deep belief network, and recurrent neural network (RNN) to identify plant species and diagnose plant diseases. They finally proved that the deep learning algorithm had broad application prospects and significant research value in the future era of smart agriculture and big data. Yang et al. (2021) studied the image recognition of wind turbine blade damage based on the deep learning model of transfer learning and ensemble learning classifier, and put forward a new method for blade damage detection based on deep learning. They tested the performance of the proposed model using the images of wind turbine blades, and they found that this model achieved better model performance than SVM, basic deep learning model, and deep learning model combined with ensemble learning method. Ntalampiras (2021) designed a twin neural network for speech-based emotion recognition through learning analogy, and the author modeled this relationship on the combined log-Mel and time-modulated spectrum space. The research results showed that the proposed framework could run under non-stationary conditions, and the model prediction could be explained by layer-by-layer investigation of the activation graph. Some related research of emotion recognition or similarity measure methods of recognition have also gained some achievements. For example, Zheng et al. (2020) they proposed a method for comparing skull similarity based on SPCA. Ben et al. (2021) they proposed a video-based facial micro-expression analysis method. In summary, the deep learning algorithm has been studied in many aspects, such as speech-based image recognition, and it has great application value in speech and image recognition.

### Graphic Visualization and Emotion Recognition

Scholars have also gained some outcomes from the research on image visualization and emotion recognition based on deep learning algorithms. Murugesan et al. (2019) conducted a comparative analysis on the performance of deep learning model in visualization and interaction and selected two real cases for the preliminary assessment to show that experts could make wise decisions on the effectiveness of different types of models. Cashman et al. (2019) investigated visual analysis through neural architecture and introduced the Fast Exploration of Model Architectures and Parameters. This visual analysis tool allowed the model builder to quickly discover the deep learning model through the exploration and rapid experiment of neural network architecture. The authors also evaluated the behavior of understanding the advantages and disadvantages of the model more minutely. Wang Y. et al. (2020) studied the deep volume synthesis network for segmentation and visualization of highly sparse and noisy image data. The authors constructed a multi-stream CNN framework to effectively learn three-dimensional volume and two-dimensional eigenvectors, respectively. Then, they explored their interdependence by back-projection of two-dimensional eigenvectors into the joint volume synthesis embedding space. Patel and Thakkar (2020) analyzed the upsurge of deep learning in computer vision applications. They found that AI technology based on deep learning was widely used in diverse fields such as network security, automobile, health, banking, retail, and financial. Wu et al. (2021) stated the great success of deep learning technology in computer vision, natural language processing, and speech recognition provided new opportunities for data visualization and analysis. Through literature survey, they found that these technologies could not only identify visual representations, but also understand analytical tasks by introducing deep learning technology into new visualization tasks. Gillmann et al. (2021) predicted medical lesions by visual multi-modal deep learning. They found that visualization could intuitively track and check the therapeutic effect of patients, and show how to improve the training database and what features can be learned by neural networks, providing useful information for medical partners. Lv et al. (2021b) utilized the deep learning algorithm for fine-grained visual computing. They integrated CNN with Network 16 model to construct a multi-level fine-grained image feature classification model, and employed the TensorFlow platform for experimental simulation. The experimental results indicated that the accuracy of the multi-level fine-grained image classification algorithm was 85.3%, and the shortest training time was 108 s, which achieved high accuracy and short training time and provided experimental reference for visual recognition.

## Experimental Method

### Signal Sampling and Establishment of the Audio-Visual Emotion Database

For continuous analog voice signals, the original signal format is not conducive to data processing and storage. Therefore, the original signal needs to be converted into discrete data through the format conversion (Narasimhan et al., 2021). For the acquisition of sound signal, excessively low sampling rate will lead to low sampling rate of model emotion recognition, but excessively high sampling rate will increase the running time of the system. Generally, the sampling rate is set to 16KHz or 8kHz. After the sampling is completed, framing and windowing are operated on the voice signal by means of uniform quantization to separate the continuous and stable voice signal into discrete finite signals. The window function used for signal windowing can be expressed as Eqs. (1) and (2).


(1) $$w\left(n\right)=\left\{ \begin{array}{cc} 1, & 0\leq n\leq \left(N-1\right)\\ 0, & \text{others} \end{array}\right.$$

(2) $$w\left(n,\alpha \right)\left\{ \begin{array}{cc} 1-\alpha -\alpha \;\cos\left(\frac{2\pi n}{N-1}\right), & 0\leq n\leq \left(N-1\right)\\ 0, & \text{others} \end{array}\right.$$

Among Eqs. (1) and (2), where *n* denotes the input of digital signals, and α refers to the windowing coefficient.

For the feature extraction of speech in the original data, firstly, the speech signal after windowing is subjected to the fast Fourier transform as shown in Eq. (3). Then, the obtained signal power spectrum needs to be filtered. Finally, the relationship between signals is extracted by discrete cosine transform, and the signal is mapped to low-dimensional space, as expressed in Eq. (4).


(3) Mel(f)=1125 ln⁡(1+f/700)

(4) $$C_{MFCC}\left(i\right)=\sqrt{2/N}\sum_{i=1}^{L}m_{l}\cos\left(\left(l-0.6\right)i\pi /L\right)$$

**FIGURE 1:**
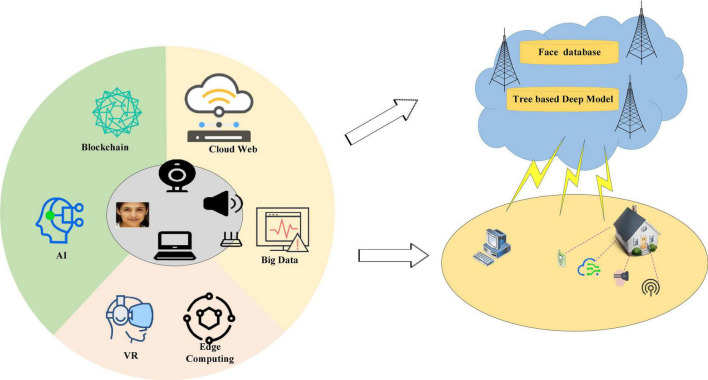
Signal collection and establishment of the experimental database.

In Eq. (3), *Mel* () represents the Mel Frequency Cepstrum Coefficient (MFCC), signifying the perception of sound, and *f* refers to the frequency. In Eq. (4), *C*_*MFCC*_ denotes the signal mapping function. Besides, the short-term energy characteristics of speech data are analyzed. *n* represents the *n*-th frame, *x*^2^_*n*_ (*m*) stands for the speech signal. The short-term energy can be calculated according to Eq. (5). Figure 1 reveals the data acquisition and establishment of the experimental database.


(5) $$E_{n}=\sum_{m=0}^{N-1}x_{n}^{2}\left(m\right)$$

### Convolution Neural Network and Facial Emotion Recognition Through Images

**FIGURE 2:**
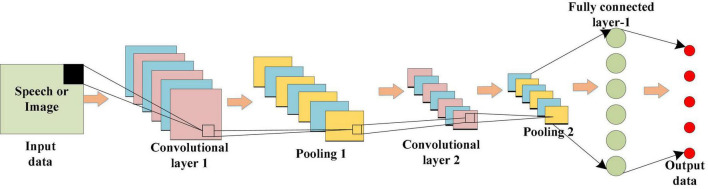
Structure of the CNN model.

As a deep neural network most commonly used to analyze visual images, CNN can greatly reduce the number of parameters in operation due to the parameter sharing mechanism, so it is widely used in image and video recognition technology. In a CNN, the input layer inputs data. For Speech or Image data, we usually convert them into a feature vector, and then input it into the neural network, and the convolution kernel in the convolution layer performs the convolution operation on the input of the upper layer and the data of this layer. Through local connection and global sharing, CNN greatly reduces the number of parameters, and enhances the learning efficiency. Through multi-layer convolution operation, the data extracted from low-level features is input into the linear rectification layer and pooling layer for down-sampling. The pooled data cannot only further reduce the network training parameters, but also strengthen the fitting degree of the model to a certain extent. Finally, the full connection layer transfers the input data to neurons, and the output layer outputs the final result. Figure 2 displays the whole operation process of CNN.

### Bi-Direction Long Short-Term Memory and Speech Emotion Recognition Mechanism

**FIGURE 3:**
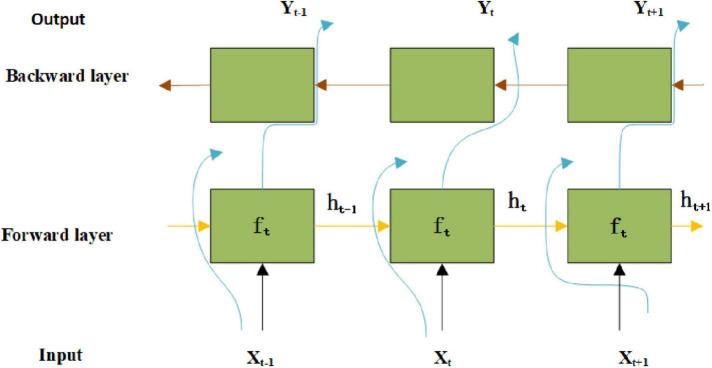
Structure of the BiLSTM network.

The Bi-direction Long Short-Term Memory (BiLSTM) network combines the advantages of Bi-directional recurrent neural network (BiRNN) and Long Short-Term Memory Network (LSTM), and has a good learning effect on the context time information in speech sequence data. Figure 3 illustrates the structure of the BiLSTM network.

In Figure 3, X_*t*_ stands for the input data at time t, Y_*t*_ represents the output data at time t. According to the circuit of the forgetting gate, the update state of unit information can be written as Eq. (6).


(6) $$f_{t}=\sigma \left(W_{f}\cdot \left[h_{t-1},x_{t}\right]+b_{f}\right)$$

In Eq. (6), where *W*_*f*_ and *b*_*f*_ represent the connection weights and bias values of the forgetting gate, respectively, and σ refers to the activation function sigmoid.

For input and output states, the state of the unit is updated by the input gate controller, and the tanh network layer compresses the hidden state information *h*_*t*–1_ and the current input information *x*_*t*_, and the result after compression is converted to the state vector *C* that can be added. A value between 0 and 1 is selected for each input value through the sigmoid network to determine which state needs to be updated according to Eqs. (7) and (8), where *W*_*i*_ and *W*_*c*_ are the connection weights of the input gate, *b*_*i*_ and *b*_*c*_ represent the bias values, and *tanh* (.) denotes the activation function tanh.


(7) $$i_{t}=\sigma \left(W_{i}\cdot \left[h_{t-1},x_{t}\right]+b_{i}\right)$$

(8) $$C_{t}=\tan h\left(W_{c}\cdot \left[h_{t-1},x_{t}\right]+b_{c}\right)$$

The current state *C*_*t*_ is the sum of the state *C*_*t*–1_ at the previous moment and the updated value *i*_t_*C_*t*_, as shown in Eq. (9).


(9) $$C_{t}=f_{t}^{*}C_{t-1}+i_{t}*C_{t}$$

For the input information at the previous time, the cells will get a new state under the action of the tanh network layer. The sigmoid network layer determines to output which part of the cell state, and the calculation process is presented in Eqs. (10) and (11), where *W*_*o*_ and *b*_*o*_ stand for the connection weights and bias values of the output gate, respectively.


(10) $$o_{t}=\sigma \left(W_{o}\cdot \left[h_{t-1},x_{t}\right]+b_{o}\right)$$

(11) $$h_{t}=O_{t}*\tan h\left(C_{t}\right)$$

here, the cross-entropy loss function is used to train the CNN-BiLSTM emotion recognition model. Eq. (12) describes the cross-entropy loss function.


(12) $$L=\frac{1}{m}\sum_{i}L_{i}=\frac{1}{m}\sum_{i}-\left[y_{i}\log\left(p_{i}\right)+\left(1-y_{i}\right)\log\left(1-p_{i}\right)\right]$$

In Eq. (12), *y*_*i*_ denotes the expected output value, which represents the label of sample *i*, and *p*_*i*_ refers to the actual output value of neurons, indicating the probability that the sample output is positive. In the process of reverse propagation, the cross-entropy loss function is derived, and the updating formulas of weight and bias is obtained, as shown in Eqs. (13) and (14).


(13) $$\frac{\partial L}{\partial W_{i}}=\frac{1}{N}\sum_{x}x_{i}\left(\sigma \left(z\right)-y_{i}\right)$$

(14) $$\frac{\partial L}{\partial b}=\frac{1}{N}\sum_{x}\left(\sigma \left(z\right)-y_{i}\right)$$

Obviously, when the error σ (*z*)-*y*_*i*_ is relatively large, the weight update is relatively fast, and when the error σ (*z*)-*y*_*i*_ is relatively small, the weight update is relatively slow. The forward propagation of the BiLSTM network can be expressed as follows:


(15) $$r_{t}=\sigma \left(W_{r}\cdot \left[h_{t-1},x_{t}\right]\right)$$

(16) $$Z_{t}=\sigma \left(W_{z}\cdot \left[h_{t-1},x_{t}\right]\right)$$

(17) $$h_{t}=\tan h\left(W_{h}\cdot \left[r_{t}*h_{t-1},x_{t}\right]\right)$$

(18) $$h_{t}=\left(1-Z_{t}\right)*h_{t-1}+Z_{t}*h_{t}$$

(19) $$y_{t}=\sigma \left(W_{o}\cdot h_{t}\right)$$

where [*h*_*t*–1_, *x*_*t*_] denotes the connection between two vectors, and * signifies the matrix multiplication. Besides, *x*_*t*_ denotes the input information of the current time, *h* (*t-1*) signifies the candidate set of the previous time, and *W*_*r*_ and *W*_*z*_ are the connection weights of the reset gate and the update gate, respectively.

After calculating the final output, the network transmission loss value of a single sample at a certain time is calculated by Eq. (20).


(20) $$E_{t}=\frac{1}{2}{\left(y_{d}-y_{t}^{o}\right)}^{2}$$

The similarity between Query input value and Source resource value is calculated, and attention value obtained through the weighted sum can be expressed as:


(21) $$Attention\left(Query,Source\right)=\sum_{i=1}^{Lx}Similarity\left(Query,Key_{i}\right)Value$$

where *Lx* = ||*source*|| represents the length of source. Eq. (22) indicates the normalized weight value α_*t*_. Moreover, the discourse level representation *c* obtained through the weighted sum, as shown in Eq. (23).


(22) $$\alpha_{t}=\frac{\exp\left(W\cdot h_{t}\right)}{\sum_{\tau =1}^{T}\exp\left(W\cdot h_{t}\right)}$$

(23) $$c=\sum_{t=1}^{T}\alpha_{t}h_{t}$$

### Emotion Recognition Method of Speech Combined With Images Based on Convolution Neural Network-Bi-Directional Long Short-Term Memory Network

**FIGURE 4:**
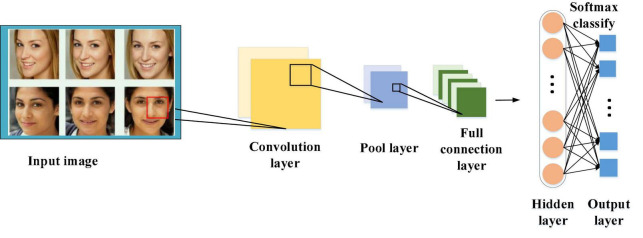
Framework of the emotion recognition algorithm of speech combined with images based on CNN-BiLSTM network.

After the pre-processing of the speech signal, the characteristic parameters are extracted from each frame to find the prosodic features of speech data (Gao et al., 2020; Han et al., 2020; Yoon et al., 2021). Figure 4 provides the overall framework of the CNN-BiLSTM network for emotion recognition of speech combined with images.

Eq. (24) indicates the short-time average zero crossing rate, and Eq. (25) represents the amplitude parameters are expressed by the waveform peak *A*_*waveform*_.


(24) $$feature_{1}=\sum_{m=-\infty }^{+\infty }Isgn\left[x\left(m\right)\right]-sgn\left[x\left(m-1\right)\right]w\left(n-m\right)$$

(25) $$feature_{2}=A_{waveform}$$

Eq. (26) signifies the nonlinear cepstrum coefficient in the Mel domain, where *f* represents the normal frequency and *f*_*Mel*_ denotes the Mel frequency.


(26) $$f_{Mel}=1125lg\left(1+\frac{f}{700}\right)$$

In the emotion recognition method of speech Combined with Images, the energy dissipation of the original data after fast Fourier transform can be calculated according to Eq. (27), and the MFCC characteristics are obtained according to Eqs. (28) and (29) through the Mel filter.


(27) $$P\left(n,k\right)=IX\left(n,k\right){|}^{2}=|\sum_{m=-0}^{N-1}x_{n}\left(m\right)e^{-j\frac{2\pi k}{N}m}{|}^{2}\left(0\leq k\leq N-1\right)$$

(28) $$feature_{3}=DCT\left(\log\left(Melfilterbank\left(P\left(n,k\right)\right)\right)\right)$$

(29) $$feature_{4}=\sqrt{a_{i}^{2}+{\left(a_{i}+\tau \right)}^{2}+{\left(a_{i}+2\tau \right)}^{2}}$$

**FIGURE 5:**
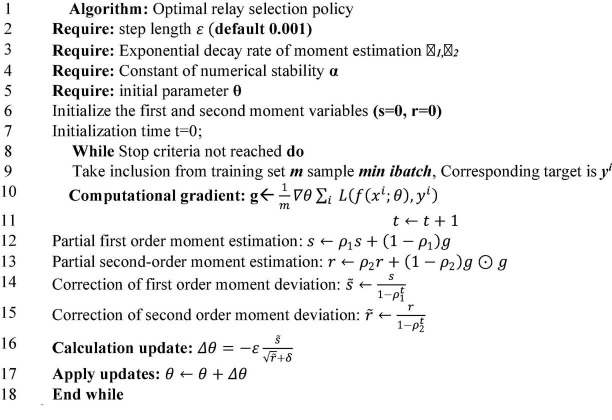
Algorithm flow.

For the nonlinear geometric characteristics of audio data, the original signal is mapped to the three-dimensional phase space to analyze the trajectory of the factor. The contours are expressed as:


(30) $$feature_{5}=\frac{\left(1,1,1\right)⊗\left(a_{i},a_{i}+\tau ,a_{i}+2\tau \right)}{\sqrt{3}}$$

(31) $$feature_{6}=\frac{\left(a_{i}-a_{i+1}\right)\cdot \left(a_{i+1}-a_{i+2}\right)}{\left|a_{i}-a_{i+1}\right|\left|a_{i+1}-a_{i+2}\right|}$$

(32) $$feature_{7}=\frac{\log{\left(R/S\right)}_{m}-\log\left(\alpha \right)}{\log\left(k\right)}$$

(33) $$feature_{8}=\frac{1}{m\tau }\frac{\ln C\left(r,m\right)}{\ln C\left(r,m+1\right)}$$

where *R* represents the range of time series, *S* refers to the standard deviation, and α denotes the coefficient. Besides, τ signifies the delay time, $\frac{\ln C\left(r,m\right)}{\ln C\left(r,m+1\right)}$ stands for the chaotic degree of time distribution probability. When processing the original data, it is necessary to package the information format of all data, denoted as *m*. In the process of signing the data, the component that obtains the forwarding right sends the data message *m* to the first unit, and the camera sends the message *m* to other components on this line for signature. Figure 5 illustrates the signature algorithm flow of other non-first units on this line to the traffic message *m* sent by the first unit.

### Experimental Analysis

For the performance evaluation of CNN-BiLSTM algorithm based on attention mechanism (Ju et al., 2020), the Berlin Emotional Database is used for the speech-based emotion recognition experiment. The experimental data is randomly divided into two groups, of which 80% is taken as the training data set, and 20% is taken as the test data set. The sampling rate is set to 16 kHz, the moving step length is set to 10 ms, and the matrix with the feature of 512 * 34 is extracted for data analysis. Moreover, the CNN-BiLSTM network architecture is realized by Keras. Adam is selected as the optimizer, the batch number is 32, and the initial learning rate is 10-4. Experimental analysis and research are carried out on 40 computers equipped with interface cameras, 3.40 GHz, 8 GB RAM, Intel (R) i5-7500, and Windows 7 operating system. The experiment is conducted in weeks. Teachers are assisted in teaching design before class. In class, learners are organized to watch online courses for learning, and meanwhile, cameras are used to collect students’ learning pictures and voice data when they answer questions. After class, images and audio data of all computers are collected.

During the experiment, the computer program automatically collects the learners’ facial expression images when the students study the course. The acquisition frequency is set to 2 frames per second, and the acquisition time is randomly set according to the class time for 5 – 10 min each time.

The CNN-BiLSTM algorithm is compared with SVM (Yu et al., 2020), Support Vector Machines-Radial Basis Function (SVM-RBF) (Sonal Singh and Kant, 2021), Extreme Learning Machine (ELM) (Wei et al., 2020), CNN (Wan et al., 2020), and BiLSTM (Wang F. K. et al., 2020) to verify the performance advantages of the CNN-BiLSTM algorithm reported here.

## Experiment Results and Discussion

### Comparison of Recognition Accuracy Performance of Different Algorithms

**FIGURE 6:**
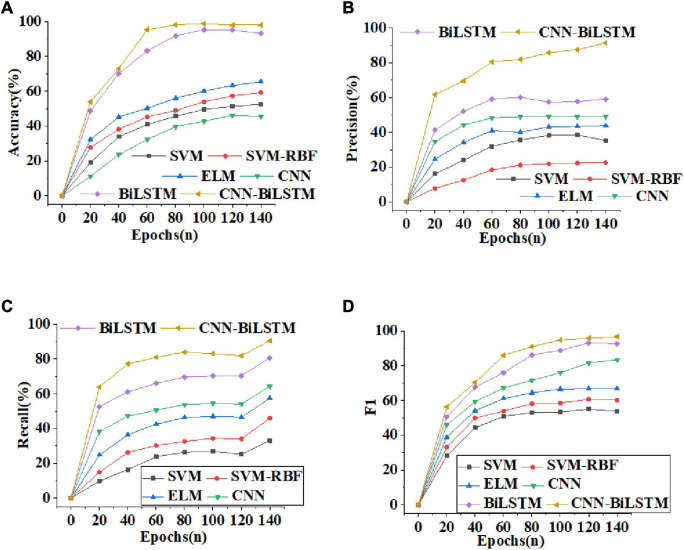
Curves of Accuracy, Precision, Recall and F1-score of different algorithms [**(A)** Accuracy; **(B)** Precision; **(C)** Recall; **(D)** F1-score].

Figure 6 illustrates the curves of Accuracy, Precision, Recall and F1-score of different algorithms. From Figure 6A, the recognition Accuracy of several comparative algorithms increases with more training periods. However, compared with other algorithms, the recognition Accuracy of the CNN-BiLSTM algorithm reported here increases faster, reaching 98.75% after 60 training periods, at least 3.15% higher than that of other algorithms. Figure 6B shows that among several algorithms, the Precision of the CNN-BiLSTM algorithm has always been optimal. When the rising trend of Precision of other algorithms slows down, the Precision of CNN-BiLSTM algorithm can still maintain rapid growth. Figure 6C shows that the Recall of CNN-BiLSTM algorithm is at least 7.13% higher than that of other algorithms. Figure 6D indicates that the F1-score of CNN-BiLSTM algorithm increases with the increase of training periods. After 100 training periods, the F1-score of the CNN-BiLSTM algorithm can reach 97.32%. Evidently, the CNN-BiLSTM algorithm has excellent performance and can achieve high accuracy of emotion recognition.

**FIGURE 7:**
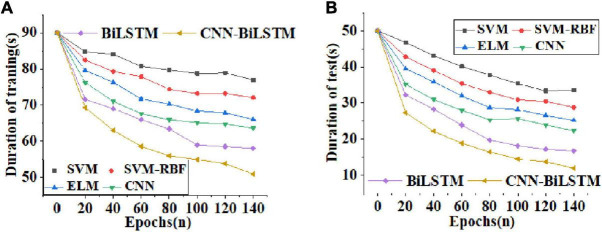
Curves of training time and test time of different algorithms [**(A)** curves of training time as the number of training periods increases; **(B)** curves of test time as the number of training periods increases].

Figure 7 reveals the curves of training time and test time of comparative algorithms.

Through the curves of training time and test time of different algorithms in Figure 7, with the increase of training periods, the training time and test time of the comparative algorithms show a downward trend. However, the CNN-BiLSTM algorithm reported here shows the largest descending gradient both in the training time (Figure 7A) and the test time (Figure 7B). After 140 training sessions, the training time of the CNN-BiLSTM algorithm is shortened to about 50 s, and the test time is shortened to 10 s. This result demonstrates that this algorithm greatly shortens the waiting time for learners’ emotion recognition and enhances the efficiency of emotion recognition.

### Comparison of Data Transmission Performance of Different Algorithms

**FIGURE 8:**
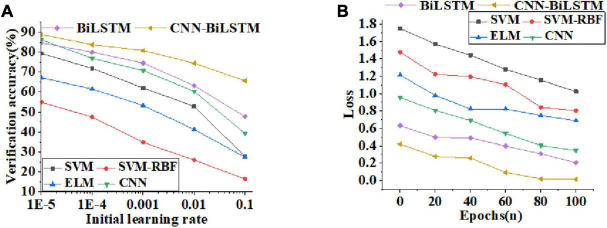
Curves of validation accuracy and function loss values of different algorithms [**(A)** curves of validation accuracy of algorithms with the increase of initial learning rate; **(B)** curves of function loss value as the number of training periods increases].

**FIGURE 9:**
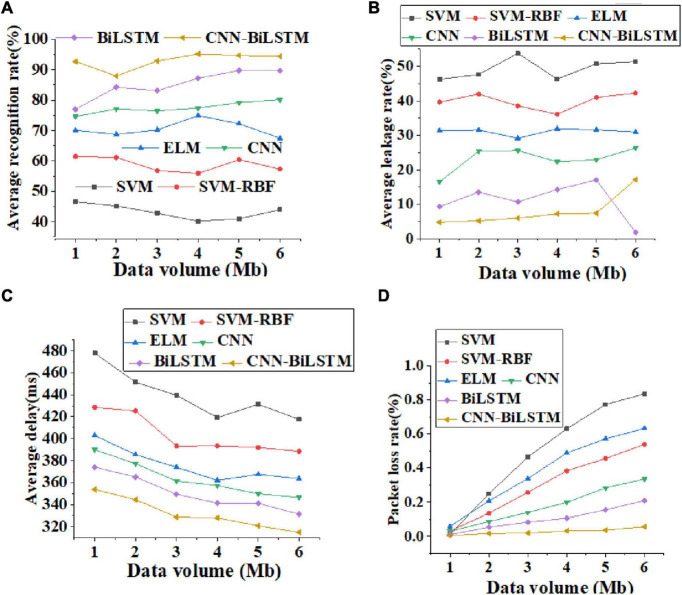
Curves of average recognition rate, average leakage rate, average delay, and packet loss rate of different algorithms [**(A)**. average recognition rate; **(B)** average leakage rate; **(C)** Average delay; **(D)** packet loss rate].

**FIGURE 10:**
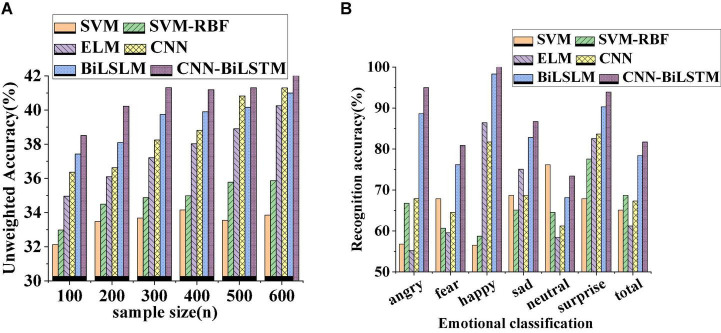
Curves of unweighted accuracy and recognition accuracy change of different algorithms [**(A)** unweighted accuracy change of different sample numbers; **(B)** changes in recognition accuracy of different emotion categories].

The data transmission performance of different algorithm models is compared and analyzed. Figure 8 displays curves of verification accuracy and function loss of different algorithms. Figure 9 illustrates curves of average recognition rate, average leakage rate, average delay, and packet loss rate of different algorithms. Figure 10 reveals curves of unweighted accuracy and recognition accuracy of different algorithms.

According to the verification accuracy curves of different algorithms in Figure 8A, the verification accuracy of CNN-BiLSTM algorithm changes little with the learning rate. With the exponential growth of the initial learning rate, the verification accuracy decreases linearly. For the change trend of function loss value with the increase of training period, Figure 8B shows that the function loss value decreases rapidly with the increase of training period. The function loss value of the CNN-BiLSTM algorithm decreases to 1.33% after 100 training periods, which excessively reduces the influence of function loss on the emotion recognition model.

Figure 9 shows the curves of average recognition rate, average leakage rate, average delay, and packet loss rate of different algorithms, where the abscissa represents the data transmission rate. Through Figure 9, with the increase in the amount of data transmitted by the algorithm, the average recognition rate of these algorithms shows an upward trend. Figure 9A shows that the recognition accuracy of the CNN-BiLSTM algorithm is not less than 90%. Figure 9B shows that the average leakage rate of data transmission of the CNN-BiLSTM algorithm does not change significantly, and the leakage rate of the CNN-BiLSTM algorithm does not exceed 15%. On the whole, the average delay decreases with the increase in transmission data. Specifically, the average delay of the CNN-BiLSTM algorithm is basically stable at about 340 ms, as shown in Figure 9C. In the packet loss rate analysis shown in Figure 9D, the SVM algorithm has the highest packet loss rate, and there may be terminal hidden problems and security risks. In addition, the CNN-BiLSTM algorithm has the lowest packet loss rate, which is not more than 5%. Therefore, from different perspectives of data transmission, the CNN-BiLSTM algorithm reported here has the characteristics of high recognition accuracy, low average leakage rate, and low delay, and shows good data transmission performance in emotion recognition.

The curves of unweighted accuracy and recognition accuracy of different algorithms presented in Figure 10 demonstrate that the unweighted accuracy of the comparative algorithms increases with the increase in the number of samples. Among them, the CNN-BiLSTM algorithm under the same number of samples has the highest unweighted accuracy. The accuracy of emotion recognition under different emotion classifications of different algorithms is shown in Figure 10B. Obviously, these algorithms have the highest recognition accuracy for happy emotion. Under the same emotional state, the recognition accuracy of CNN-BiLSTM algorithm is the highest among several algorithms, indicating that CNN-BiLSTM algorithm has superior performance and is competent to be used as an application model for emotion recognition and graphical visualization.

## Conclusion

With the rapid development of Internet technology, the existing learning methods have begun to change. The intelligent learning environment based on IoT characterized by the digital high-end form has gradually become the principal way of learning knowledge. Based on the emotional interaction theory of learners in an intelligent learning environment, the CNN-BiLSTM algorithm is utilized to realize the real-time recognition of learners’ emotions, to promote the relaxing and effective learning of learners. The experimental results show that the accuracy of CNN-BiLSTM algorithm reported here reaches 98.75%, which is at least 3.15% higher than that of other comparative algorithms, and the recall rate is at least 7.13% higher than that of other algorithms. Besides, the recognition accuracy is not less than 90%, which can achieve good recognition results. However, there are still some inevitable shortcomings. Firstly, the expression image database about learning pictures and learners should continue to expand, since the expression image database used here contains insufficient image data. Secondly, the real-time emotion recognition algorithm for learning images based on CNN-BiLSTM algorithm should be optimized to further improve the accuracy and efficiency of the algorithm.

## Data Availability Statement

The original contributions presented in the study are included in the article/supplementary material, further inquiries can be directed to the corresponding author.

## Ethics Statement

The individual(s) provided their written informed consent for the publication of any identifiable images or data presented in this article.

## Author Contributions

XL contributed to conception and design of the study and wrote the first draft of the manuscript. The author contributed to manuscript revision, read, and approved the submitted version.

## Conflict of Interest

The author declares that the research was conducted in the absence of any commercial or financial relationships that could be construed as a potential conflict of interest.

## Publisher’s Note

All claims expressed in this article are solely those of the authors and do not necessarily represent those of their affiliated organizations, or those of the publisher, the editors and the reviewers. Any product that may be evaluated in this article, or claim that may be made by its manufacturer, is not guaranteed or endorsed by the publisher.
